# An international multicenter study comparing COVID‐19 omicron outcomes in patients with hematological malignancies treated with obinutuzumab versus rituximab

**DOI:** 10.1002/cam4.6997

**Published:** 2024-02-24

**Authors:** Tali Shafat, Daniel Grupel, Tzvika Porges, Ran Abuhasira, Ana Belkin, Ofir Deri, Yonatan Oster, Shadi Zahran, Ehud Horwitz, Netanel A. Horowitz, Hazim Khatib, Marjorie Vieira Batista, Anita Cassoli Cortez, Tal Brosh‐Nissimov, Yafit Segman, Linor Ishay, Regev Cohen, Alaa Atamna, Amy Spallone, Roy F. Chemaly, Juan Carlos Ramos‐Ramos, Michal Chowers, Evgeny Rogozin, Noga Carmi Oren, Şiran Keske, Orit Wolfovitz Barchad, Lior Nesher, the European Society of Clinical Microbiology and Infectious Diseases (ESCMID) Study Group for Respiratory Viruses (ESGREV)

**Affiliations:** ^1^ Infectious Diseases Institute, Soroka University Medical Center, and the Faculty of Health Sciences Ben‐Gurion University of the Negev Beer‐Sheva Israel; ^2^ Clinical Research Center, Soroka University Medical Center, and the Faculty of Health Sciences Ben‐Gurion University of the Negev Beer‐Sheva Israel; ^3^ Department of Infectious Diseases, Infection Control and Employee Health The University of Texas MD Anderson Cancer Center Houston Texas USA; ^4^ European Society of Clinical Microbiology and Infectious Diseases (ESCMID) Study Group for Respiratory Viruses (ESGREV) Basel Switzerland; ^5^ Department of Clinical Microbiology and Infectious Diseases Hadassah Medical Center Jerusalem Israel; ^6^ Faculty of Medicine Hebrew University Jerusalem Israel; ^7^ Hematology Department, Soroka University Medical Center, and the Faculty of Health Sciences Ben‐Gurion University of the Negev Beer‐Sheva Israel; ^8^ Internal Medicine D and Infectious Diseases Unit Sheba Medical Center Ramat‐Gan Israel; ^9^ Sackler Faculty of Medicine Tel Aviv University Ramat‐Aviv Israel; ^10^ Internal Medicine T Sheba Medical Center Ramat‐Gan Israel; ^11^ Department of Hematology and Bone Marrow Transplantation Rambam Health Care Campus Haifa Israel; ^12^ Department of Infectious Diseases AC Camargo Cancer Center São Paulo São Paulo Brazil; ^13^ Department of Hematology and Cell Therapy AC Camargo Cancer Center São Paulo São Paulo Brazil; ^14^ Infectious Diseases Unit Samson Assuta Ashdod University Hospital Ashdod Israel; ^15^ The Faculty of Health Sciences Ben‐Gurion University of the Negev Beer‐Sheva Israel; ^16^ Hematology Institute Samson Assuta Ashdod University Hospital Ashdod Israel; ^17^ Rappaport Faculty of Medicine Technion Haifa Israel; ^18^ Hillel Yaffe Medical Center Hadera Israel; ^19^ Infectious Diseases Unit, Rabin Medical Center Beilinson Hospital Petah Tikva Israel; ^20^ Infectious Disease Unit Internal Medicine Service. CIBERINFEC. Hospital Universitario La Paz Madrid Spain; ^21^ Meir Medical Centre Kfar Saba Israel; ^22^ Sackler Faculty of Medicine Tel Aviv University Tel Aviv Israel; ^23^ Infectious Diseases unit Shamir (Assaf Harofeh) Medical Center Be'er Ya'akov Israel; ^24^ Department of Infectious Diseases VKV American Hospital Istanbul Turkey; ^25^ Infectious Disease Unit Shaare Zedek Medical Center Jerusalem Israel

**Keywords:** anti‐CD20 monoclonal antibodies, COVID‐19, hematological malignancies, obinutuzumab, rituximab

## Abstract

**Objectives:**

Hematological malignancy (HM) patients treated with anti‐CD20 monoclonal antibodies are at higher risk for severe COVID‐19. A previous single‐center study showed worse outcomes in patients treated with obinutuzumab compared to rituximab. We examined this hypothesis in a large international multicenter cohort.

**Methods:**

We included HM patients from 15 centers, from five countries treated with anti‐CD20, comparing those treated with obinutuzumab (O‐G) to rituximab (R‐G) between December 2021 and June 2022, when Omicron lineage was dominant.

**Results:**

We collected data on 1048 patients. Within the R‐G (*n* = 762, 73%), 191 (25%) contracted COVID‐19 compared to 103 (36%) in the O‐G. COVID‐19 patients in the O‐G were younger (61 ± 11.7 vs. 64 ± 14.5, *p* = 0.039), had more indolent HM diagnosis (aggressive lymphoma: 3.9% vs. 67.0%, *p* < 0.001), and most were on maintenance therapy at COVID‐19 diagnosis (63.0% vs. 16.8%, *p* < 0.001). Severe‐critical COVID‐19 occurred in 31.1% of patients in the O‐G and 22.5% in the R‐G. In multivariable analysis, O‐G had a 2.08‐fold increased risk for severe‐critical COVID‐19 compared to R‐G (95% CI 1.13–3.84), adjusted for Charlson comorbidity index, sex, and tixagevimab/cilgavimab (T‐C) prophylaxis. Further analysis comparing O‐G to R‐G demonstrated increased hospitalizations (51.5% vs. 35.6% *p* = 0.008), ICU admissions (12.6% vs. 5.8%, *p* = 0.042), but the nonsignificant difference in COVID‐19‐related mortality (*n* = 10, 9.7% vs. *n* = 12, 6.3%, *p* = 0.293).

**Conclusions:**

Despite younger age and a more indolent HM diagnosis, patients receiving obinutuzumab had more severe COVID‐19 outcomes than those receiving rituximab. Our findings underscore the need to evaluate the risk–benefit balance when considering obinutuzumab therapy for HM patients during respiratory viral outbreaks.

## Graphical abstract

An international multicancer study of 1048 hematological cancer patients, shows those on obinutuzumab faced higher risks of severe omicron COVID‐19 outcomes than those on rituximab, despite being younger and with fewer comorbidities.

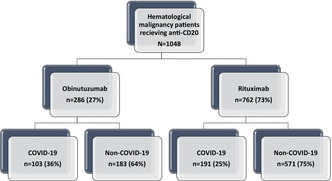

## 1 INTRODUCTION

Patients with hematological malignancies (HM) are at a higher risk for complications and mortality due to COVID‐19.^1^ Within these patients, those receiving anti‐CD20 therapy are especially at risk for persistent disease, relapse, respiratory failure, and death.^2^, ^3^ During the pandemic, nearing the end of 2021, the dominant SARS‐CoV‐2 variants were the Omicron lineage subvariants,^4^, ^5^ which are generally characterized by a lower case fatality rate, lower propensity for complications but substantially higher transmissibility^6^; however, patients with HM remained at his risk for complications and mortality.^7^, ^8^ Antivirals such as Nirmatrelvir‐Ritonavir, Remdesivir, Molnupiravir, and monoclonal antibodies targeting SARS‐CoV‐2 became widely available during this period. These treatments benefit patients with HM who cannot mount an effective humoral response to SARS‐CoV‐2 infection or develop meaningful immunity following SARS‐CoV‐2 vaccines.^9^, ^10^

In recent years, anti‐CD20 treatments, rituximab, and obinutuzumab, have been widely used for HM. Alongside their beneficial anti‐lymphoma effects, these antibodies neutralize B cells and suppress the humoral response to viral replication,^11^ resulting in a higher risk for severe COVID‐19.^2^ Obinutuzumab is a novel type 2 anti‐CD20 that, when compared with rituximab, causes enhanced antibody‐dependent cell‐mediated cytotoxicity, which induces higher levels of direct cell death.^12^, ^13^, ^14^ Obinutuzumab is used (alone or with chemotherapy) mainly for previously untreated or relapsed follicular lymphoma (FL) or chronic lymphocytic leukemia (CLL). Obinutuzumab‐based immunochemotherapy and maintenance resulted in more prolonged progression‐free survival (PFS) for patients with advanced‐stage FL, but there were more adverse events, including infections.^15^

A previous single‐center study^16^ demonstrated that HM patients who had COVID‐19 while on treatment with obinutuzumab rather than rituximab had worse clinical outcomes, including higher severe‐critical illness (35% compared to 7% *p* = 0.017), hospitalization rate (60% compared to 25.9% *p* = 0.019), and all‐cause mortality (3/20 vs. 0/27). This study aims to examine and validate this concept in a large international cohort.

## 2 METHODS

### 2.1 Study design and participants

We conducted a retrospective international multicenter cohort study across 15 centers from Israel, Turkey, Spain, Brazil, and the USA. The study period was between December 1, 2021, and June 30, 2022, when the Omicron lineage variants were predominant. We included patients with HM treated with either obinutuzumab or rituximab starting 6 months before and throughout the study. COVID‐19 diagnosis was established based on a positive SARS‐CoV‐2 PCR test; all centers tested individuals who exhibited symptoms or were part of exposure tracing during epidemiological investigations. There was no formal screening for asymptomatic patients. We excluded patients diagnosed with COVID‐19 before their first anti‐CD20 treatment.

### 2.2 Hematological malignancies definitions

We defined aggressive hematological malignancy as patients with B‐cell NHL, including DLBCL, mantle cell lymphoma, and high‐grade lymphoma, treated with RCHOP or CHOP‐like regimens. Indolent lymphoma included follicular lymphoma and marginal zone lymphoma. Patients in remission had a negative PET‐CT scan before contracting COVID‐19. The relapsed/refractory group included patients who received anti‐CD20 therapy as a second or later line of treatment.

### 2.3 Study outcomes

The primary outcome was the development of severe or critical COVID‐19, defined as patients with SpO2 ≤94% on room air, including those on supplemental oxygen, oxygen through a high‐flow device, mechanical ventilation, or extracorporeal membrane oxygenation (ECMO)^17^. Secondary outcomes were hospitalization, ICU admission, COVID‐19‐related mortality, and all‐cause mortality.

### 2.4 Data collection

Baseline characteristics, including age, sex, Charlson comorbidity index (CCI), and medical history, were collected for all participants. We noted the use of tixagevimab‐cilgavimab (T‐C) prophylaxis and COVID‐19 vaccination status (number of doses received) when available. We recorded the hematological malignancy diagnosis, previous treatments, and the stage of HM treatments (induction or maintenance) when diagnosed with COVID‐19. We collected COVID‐19‐related information, including COVID‐19 severity (asymptomatic‐mild–moderate or severe‐critical) and the need for respiratory support (none, nasal cannula, facemasks with oxygen reservoir bag, high flow nasal cannula, noninvasive ventilation, invasive mechanical ventilation, and ECMO). We recorded the time from the last anti‐CD20 therapy to the COVID‐19 diagnosis and the duration of hospitalization.

Study data were collected and managed using REDCap (Research Electronic Data Capture) tools hosted at Ben‐Gurion University.^18^ REDCap is a secure, web‐based software platform that supports data capture for research studies. This study followed the STROBE guidelines^19^ and complied with ethical and Good Clinical Practice guidelines and regulations. The study was approved, and a waiver of informed consent was granted by the Soroka Ethics in research committee (SOR‐22‐0198) and by each participating institution's committee. Patient information was de‐identified and kept confidential throughout the study.

### 2.5 Statistical analysis

Continuous variables were presented as mean ± standard deviation or median and interquartile range if non‐normally distributed, while categorical data were presented as total patients (percentage of all patients). Continuous variables were compared using *t*‐tests or the Mann–Whitney test, and categorical data were analyzed using chi‐square (*χ* ^2^) or Fisher's exact tests. Multivariable analysis for severe COVID‐19 and secondary outcomes was performed using a logistic regression model. Variables with clinical significance were incorporated in the model as comorbidities and age were shown to be related to COVID‐19 outcomes in this population,^1^ as well as T‐C prophylaxis.^20^ Survival analysis was conducted using Kaplan–Meier's survival table and curve. A subgroup analysis was conducted specifically on patients with indolent lymphoma/CLL. Multivariate analysis within this group was conducted using logistic regression. Independent variables incorporated in this model were also selected upon a clinical basis, and included CCI and HM treatment stage at COVID‐19 diagnosis.^1^ The statistical software SPSS version 25 (IBM Corp Armonk, NY, USA) was used, and a two‐tailed *p* ≤0.05 was considered significant.

## 3 RESULTS

**FIGURE 1:**
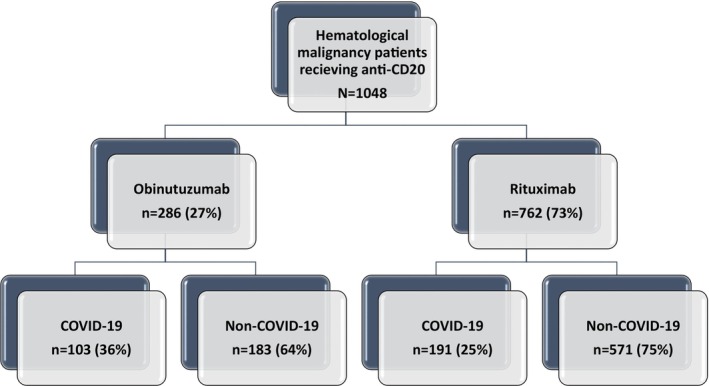
Flowchart of the study population.

During the study period, a total of 1048 patients with HM received at least one dose of anti‐CD20, of which 762 (73%) received rituximab and 286 (27%) received obinutuzumab (Figure 1). Characteristics of the population corresponding to the anti‐CD20 type are shown in Table 1. The majority of patients were treated in Israel (Table S1a,b). During the study period, 28.1% of the cohort (294/1048) contracted SARS‐CoV‐2, 191 (25.1%) in the rituximab group (R‐G), and 103 (36.0%) in the obinutuzumab group (O‐G). Median follow‐up duration was 6.9 months (IQR 4.5–9.6).

**TABLE 1:** Characteristics of the study population according to anti‐CD20 type.

Variables	R‐G (*n* = 762)	O‐G (*n* = 286)	Total (*n* = 1048)	*p* value (R‐G vs. O‐G)
Baseline characteristics				
Age (mean ± SD)	64.0 ± 15.4	60.0 ± 12.5	62.9 ± 14.8	<0.001
Sex, female, *n* (%)	341 (45.1)	131 (46.0)	472 (45.3)	0.804
COVID‐19 vaccination status, *n* (%) (*n* = 868)				
0–2 doses	214 (33.9)	83 (35.0)	297 (34.2)	0.759
3–5 doses	417 (66.1)	154 (65.0)	571 (65.8)	
Tixagevimab‐cilgavimab prophylaxis, *n* (%) (*n* = 878)	156 (24.1)	69 (29.7)	225 (25.6)	0.094
Hematological malignancy status				
Hematologic diagnosis, *n* (%)				
Aggressive lymphoma	466 (61.2)	17 (5.9)	483 (46.1)	<0.001
Indolent lymphoma	259 (34.0)	210 (73.4)	469 (44.8)	
Chronic lymphocytes leukemia	37 (4.8)	59 (20.7)	96 (9.1)	
s/p CAR‐T, *n* (%)	32 (4.2)	5 (1.7)	37 (3.5)	0.055
s/p HSCT, *n* (%)	49 (6.4)	3 (1.0)	52 (5.0)	<0.001
Anti‐CD20 as first‐line therapy, *n* (%)	680 (89.8)	254 (89.1)	934 (89.6)	0.739
Outcomes				
All‐cause mortality, *n* (%)	42 (5.5)	17 (5.9)	59 (5.6)	0.787
Follow up duration, months (median, IQR)	6.9, 4.0–6.9	6.9, 5.6–6.9	6.9, 4.5–6.9	<0.001

Abbreviations: CAR‐T, Chimeric antigen receptor (CAR) T‐cell therapy; COVID‐19, SARS‐CoV‐2 disease 2019; HSCT, hematopoietic stem cell transplantation; IQR, interquartile range; O‐G, Obinutuzumab‐treated group; R‐G, Rituximab‐treated group.

The characteristics, COVID‐19 severity, and outcomes of those infected with COVID‐19 are shown in Table 2. Comparing patients in the O‐G to R‐G, more O‐G patients required oxygen supplementation or respiratory support (35.0% vs. 23.6%, *p* = 0.037), had a higher hospitalization rate (53/103, 51.5% vs. 68/191, 35.6%, *p* = 0.008), a higher proportion of ICU admissions (13/103, 12.6% vs. 11/191, 5.8%, *p* = 0.042), and a higher need for invasive mechanical ventilation (11/103, 10.7% vs. 4/191, 2.1%, *p* = 0.001). Conversely, all‐cause and COVID‐19‐related mortality were similar between the two groups. Among the O‐G, there were 10 deaths during the follow‐up period, all related to COVID‐19, while among the R‐G, 18 deaths occurred, with 12 related to COVID‐19.

**TABLE 2:** Characteristics, COVID‐19 severity, and outcomes of patients infected with COVID‐19 according to anti‐CD20 type.

Variables	R‐G (*n* = 191)	O‐G (*n* = 103)	Total (*n* = 294)	*p* value (R‐G vs. O‐G)
Baseline characteristics				
Age (mean ± SD)	64.5 ± 14.5	61.0 ± 11.7	63.3 ± 13.7	0.039
Age ≥65 years, *n* (%)	106 (56.7)	43 (41.7)	149 (51.4)	0.015
Sex, female, *n* (%)	91 (47.6)	52 (51.0)	143 (48.8)	0.586
Charlson comorbidity index (median, IQR)	5, 4–7	5, 3–6	5, 3–7	0.026
Charlson comorbidity index ≥4, *n* (%)	142 (75.9)	71 (68.9)	213 (73.4)	0.196
Chronic ischemic heart disease, *n* (%)	15 (7.9)	7 (6.8)	22 (7.5)	0.742
Heart failure, *n* (%)	11 (5.8)	1 (1.0)	12 (4.1)	0.048
s/p stroke, *n* (%)	9 (4.7)	4 (3.9)	13 (4.4)	0.742
Dementia, *n* (%)	8 (4.2)	1 (1.0)	9 (3.1)	0.127
Diabetes mellitus, *n* (%)	32 (16.8)	20 (19.4)	52 (17.7)	0.568
Diabetes mellitus with organ damage, *n* (%)	4 (2.1)	3 (2.9)	7 (2.4)	0.699
Chronic obstructive pulmonary disease, *n* (%)	9 (4.7)	8 (7.8)	17 (5.8)	0.284
Tixagevimab‐cilgavimab prophylaxis, *n* (%) (*n* = 265)	19 (10.9)	14 (15.6)	33 (12.5)	0.273
COVID‐19 vaccination status, *n* (%) (*n* = 268)				
0–2 doses	62 (34.6)	32 (36.0)	94 (35.1)	0.831
3–5 doses	117 (65.4)	57 (64.0)	174 (64.9)	
Hematological malignancy status				
Hematologic diagnosis, *n* (%)				
Aggressive lymphoma	128 (67.0)	4 (3.9)	132 (44.9)	<0.001
Indolent lymphoma	53 (27.7)	74 (71.8)	127 (43.2)	
Chronic lymphocytes leukemia	10 (5.3)	25 (24.3)	35 (11.9)	
s/p CAR‐T, *n* (%)	6 (3.2)	2 (1.9)	8 (2.7)	0.542
s/p HCT, *n* (%)	17 (8.9)	2 (1.9)	19 (6.5)	0.020
Anti‐CD20 as first‐line therapy, *n* (%)	169 (89.4)	91 (88.3)	260 (89.0)	0.780
Therapy stage at COVID‐19 infection, *n* (%) (*n* = 285)				
Induction	154 (83.2)	37 (37.0)	191 (67.0)	<0.001
Maintenance	31 (16.8)	63 (63.0)	94 (33.0)	
COVID‐19 disease				
COVID‐19 severity, *n* (%)				
Asymptomatic‐mild–moderate	148 (77.5)	71 (68.9)	219 (74.5)	0.108
Severe‐critical	43 (22.5)	32 (31.1)	75 (25.5)	
Respiratory support, *n* (%)				
None	146 (76.4)	67 (65.0)	213 (72.4)	0.037
Nasal prongs	21 (11.0)	11 (10.7)	32 (10.9)	0.934
Facemasks with oxygen reservoir bag	7 (3.7)	2 (1.9)	9 (3.1)	0.413
High flow nasal prongs	7 (3.7)	5 (4.9)	12 (4.1)	0.623
Noninvasive ventilation	2 (1.0)	2 (1.9)	4 (1.4)	0.614
Mechanical ventilation	4 (2.1)	11 (10.7)	15 (5.1)	0.001
ECMO	0 (0)	1 (1.0)	1 (0.3)	0.350
Time from last anti‐CD20 to COVID‐19 diagnosis, days (median, IQR)	56, 20–162	46, 21–145	49, 20–156	0.656
Time from last anti‐CD20 to COVID‐19 diagnosis, *n* (%)				
<30 days	70 (36.7)	38 (36.9)	108 (36.7)	0.293
30–90 days	47 (24.6)	33 (32.0)	80 (27.2)	
>90 days	74 (38.7)	32 (31.1)	106 (36.1)	
Hospitalizations, *n* (%)	68 (35.6)	53 (51.5)	121 (41.2)	0.008
ICU hospitalization, *n* (%)	11 (5.8)	13 (12.6)	24 (8.2)	0.042
Hospital LOS (median, IQR) (*n* = 101)	6, 4–11	8, 4–14	7, 4–13	0.271
Treatment, *n* (%)				
Remdesivir	53 (27.7)	32 (31.1)	85 (28.9)	0.549
Nirmatrelvir/Ritonavir	51 (26.7)	28 (27.2)	79 (26.9)	0.929
Molnupiravir	13 (6.8)	7 (6.8)	20 (6.8)	0.997
Any antiviral	108 (56.5)	64 (62.1)	172 (58.5)	0.353
Glucocorticoids	71 (37.2)	45 (43.7)	116 (39.5)	0.275
Baricitinib	12 (6.3)	11 (10.7)	23 (7.8)	0.180
Convalescence plasma	17 (8.9)	18 (17.5)	35 (11.9)	0.030
Tixagevimab‐cilgavimab	11 (5.8)	13 (12.6)	24 (8.2)	0.040
Outcomes				
COVID‐19‐related mortality, *n* (%)	12 (6.3)	10 (9.7)	22 (7.5)	0.293
All‐cause mortality, *n* (%)	18 (9.4)	10 (9.7)	28 (9.5)	0.937
Follow‐up duration, months (median, IQR)	6.9, 5.4–6.9	6.9, 5.7–6.9	6.9, 5.6–6.9	0.600

Abbreviations: CAR‐T, Chimeric antigen receptor (CAR) T‐cell therapy; COVID‐19, SARS‐CoV‐2 disease 2019; ECMO, extracorporeal membrane oxygenation; HSCT, hematopoietic stem cell transplantation; ICU, intensive care unit; IQR, interquartile range; LOS, length of stay; O‐G, Obinutuzumab‐treated group; R‐G, Rituximab‐treated group.

### 3.1 Severe to critical COVID‐19

A higher proportion of patients in the O‐G were diagnosed with severe to critical COVID‐19 compared to the R‐G; these differences were not statistically significant on univariate analysis (31.1% vs. 22.5%, *p* = 0.108) (Table 2). However, in the multivariable analysis, the patients on obinutuzumab had a 2.08‐fold increased risk for the primary outcome of severe to critical COVID‐19 compared to rituximab, adjusted for CCI, sex, and T‐C prophylaxis (OR = 2.08, 95% CI 1.13–3.84) (Table 3). Each point increase in CCI was associated with a higher risk of severe to critical COVID‐19 (OR = 1.22, 95% CI 1.09–1.37). T‐C prophylaxis was associated with a lower risk of severe COVID‐19 (OR = 0.32, 95% CI 0.10–0.99). Anti‐SARS‐CoV‐2 vaccination status of 3–4 doses (in comparison to 0–2 doses) was not associated with lower risk for severe‐critical COVID‐19 (a multivariable model including this variable is shown in Table S2).

**TABLE 3:** Multivariable analysis demonstrating the association between anti‐CD20 treatment, patient characteristics, and COVID‐19 outcomes.

	Severe‐critical COVID‐19^a^	Hospitalization^a^	ICU admission^a^	Mechanical ventilation^a^
Obinutuzumab therapy (with rituximab as reference)	2.08 (1.13–3.84)	2.48 (1.44–4.26)	3.06 (1.16–8.05)	6.10 (1.68–22.20)
Charlson comorbidity index (for each additional point)	1.22 (1.09–1.37)	1.12 (1.01–1.24)	1.15 (0.96–1.37)	1.18 (0.94–1.48)
Sex, female	0.95 (0.53–1.70)	1.11 (0.66–1.84)	0.98 (0.39–2.49)	0.58 (0.18–1.88)
Tixagevimab‐cilgavimab prophylaxis	0.32 (0.10–0.99)	0.45 (0.19–1.04)	0.29 (0.04–2.33)	0.39 (0.04–3.35)

Abbreviations: COVID‐19, SARS‐CoV‐2 disease 2019; ICU, intensive care unit.

^a^ Each model included the following parameters: anti‐CD20 type (obinutuzumab/rituximab), Charlson comorbidity index, sex, and tixagevimab‐cilgavimab prophylaxis.

For secondary outcomes, in a multivariable analysis adjusted for Charlson comorbidity index, sex and tixagevimab‐cilgavimab prophylaxis, obinutuzumab therapy was associated with increased risk of hospitalization OR = 2.48 (95% CI 1.44–4.26), ICU admission OR = 3.06 (95% CI 1.16–8.05), and mechanical ventilation OR = 6.10 (95% CI 1.68–22.20) (Table 3).

### 3.2 Subgroup analysis of patients with indolent lymphoma or CLL

Within the 469 patients with indolent lymphoma or CLL, we conducted a subgroup analysis of 162 patients who acquired COVID‐19, which showed that O‐G compared to R‐G were younger (61.7 ± 11.0 vs. 67.9 ± 13.5 years *p* = 0.002), were in the maintenance therapy phase during COVID‐19 diagnosis (64.6% vs. 46.7%, *p* = 0.028), and had a lower CCI (69.7% vs. 85.7% with CCI≥4, *p* = 0.020) (Table 4). While the overall proportion of severe‐critical COVID‐19 was similar between the two groups (31.3% O‐G vs. 30.2% R‐G, *p* = 0.877), the O‐G had a higher incidence of critical disease indicators. Specifically, 11 patients (11.1%) in the O‐G required invasive mechanical ventilation compared to only one patient (1.6%) in the R‐G (*p* = 0.024). Additionally, there were 13 ICU admissions (13.1%) in the O‐G group compared to three admissions (4.8%) in the R‐G (*p* = 0.087). In the multivariable analysis, adjusting for CCI and therapy stage at COVID‐19 diagnosis, obinutuzumab treatment was found to be independently associated with a 4.6‐fold increased risk of ICU admission (OR 4.62, 95% CI 1.14–18.67), compared to rituximab (Table S3). Outcomes of COVID‐19‐infected patients during the induction and maintenance treatment phases separately, are presented in Table S4a,b.

**TABLE 4:** Characteristics of positive COVID‐19 patients with indolent lymphoma/chronic lymphocytic leukemia.

Variables	R‐G (*n* = 63)	O‐G (*n* = 99)	Total (*n* = 162)	*p* value (R‐G vs. O‐G)
Baseline characteristics				
Age (mean ± SD)	67.9 ± 13.5	61.7 ± 11.0	64.1 ± 12.4	0.002
Age ≥65 years, *n* (%)	43 (68.3)	43 (43.4)	86 (53.1)	0.002
Sex, female, *n* (%)	39 (61.9)	49 (50.0)	88 (54.7)	0.139
Charlson comorbidity index, (median, IQR)	5, 4–7	5, 3–6	5, 4–6	0.006
Charlson comorbidity index ≥4, *n* (%)	54 (85.7)	69 (69.7)	123 (75.9)	0.020
Chronic ischemic heart disease *n* (%)	6 (9.5)	7 (7.1)	13 (8.0)	0.575
Heart failure, *n* (%)	3 (4.8)	1 (1.0)	4 (2.5)	0.300
s/p stroke, *n* (%)	5 (7.9)	4 (4.0)	9 (5.6)	0.291
Dementia, *n* (%)	3 (4.8)	1 (1.0)	4 (2.5)	0.300
Diabetes mellitus, *n* (%)	6 (9.6)	19 (19.2)	25 (15.4)	0.097
Chronic obstructive pulmonary disease, *n* (%)	4 (6.3)	8 (8.1)	12 (7.4)	0.682
Tixagevimab‐cilgavimab prophylaxis, *n* (%) (*n* = 146)	6 (10.0)	14 (16.3)	20 (13.7)	0.278
COVID‐19 vaccination status, *n* (%) (*n* = 145)				
0–2 doses	19 (31.7)	30 (35.3)	49 (33.8)	0.649
3–5 doses	41 (68.3)	55 (64.7)	96 (66.2)	
Hematological malignancy status				
Hematologic diagnosis, *n* (%)				
Indolent lymphoma	53 (84.1)	74 (74.7)	127 (78.4)	0.157
Chronic lymphocytes leukemia	10 (15.9)	25 (25.3)	35 (21.6)	
s/p HSCT, *n* (%)	2 (3.2)	1 (1.0)	3 (1.9)	0.561
Anti‐CD20 as first‐line therapy, *n* (%)	53 (85.5)	89 (89.9)	142 (88.2)	0.398
Therapy stage at COVID‐19 diagnosis, *n* (%) (*n* = 156)				
Induction	32 (53.3)	34 (35.4)	66 (42.3)	0.028
Maintenance	28 (46.7)	62 (64.6)	90 (57.7)	
COVID‐19 disease				
COVID severity, *n* (%)				
Asymptomatic‐mild–moderate	38 (71.7)	50 (67.6)	112 (69.1)	0.877
Severe‐critical	19 (30.2)	31 (31.3)	50 (30.9)	
Respiratory support, *n* (%)				
None	43 (68.3)	64 (64.6)	107 (66.0)	0.636
Nasal prongs	12 (19.0)	11 (11.1)	23 (14.2)	0.158
Facemasks with oxygen reservoir bag	3 (4.8)	2 (2.0)	5 (3.1)	0.378
High flow nasal prongs	1 (1.6)	4 (4.0)	5 (3.1)	0.649
Noninvasive ventilation	1 (1.6)	2 (2.0)	3 (1.9)	1.000
Mechanical ventilation	1 (1.6)	11 (11.1)	12 (7.4)	0.024
ECMO	0 (0)	1 (1.0)	1 (0.6)	1.000
Time from last anti‐CD20 to COVID‐19 infection, *n* (%)				
<30 days	18 (28.6)	36 (36.4)	54 (33.3)	0.167
30–90 days	16 (25.4)	32 (32.3)	48 (29.6)	
>90 days	29 (46.0)	31 (31.3)	60 (37.0)	
Hospitalizations, *n* (%)	25 (39.7)	50 (50.5)	75 (46.3)	0.178
ICU hospitalization, *n* (%)	3 (4.8)	13 (13.1)	16 (9.9)	0.087
Hospital LOS (median, IQR) (*n* = 65)	6, 3–10	8, 4–14	7, 4–12	0.310
Treatment, *n* (%)				
Remdesivir	23 (36.5)	29 (29.3)	52 (32.1)	0.338
Nirmatrelvir/Ritonavir	11 (17.5)	28 (28.3)	39 (24.1)	0.116
Molnupiravir	8 (12.7)	7 (7.1)	15 (9.3)	0.228
Any antiviral	38 (60.3)	61 (61.6)	99 (61.1)	0.869
Glucocorticoids	29 (46.0)	43 (43.4)	72 (44.4)	0.746
Baricitinib	4 (6.3)	11 (11.1)	15 (9.3)	0.308
Convalescence plasma	9 (14.3)	18 (18.2)	27 (16.7)	0.517
Tixagevimab‐cilgavimab	5 (7.9)	13 (13.1)	18 (11.1)	0.305
Outcomes				
COVID‐19‐related mortality, *n* (%)	4 (6.5)	9 (9.1)	13 (8.1)	0.550
All‐cause mortality, *n* (%)	5 (7.9)	9 (9.1)	14 (8.6)	0.799

Abbreviations: COVID‐19, SARS‐CoV‐2 disease 2019; ECMO, extracorporeal membrane oxygenation; HSCT, hematopoietic stem cell transplantation; ICU, intensive care unit; IQR, interquartile range; LOS, length of stay; O‐G, Obinutuzumab‐treated group; R‐G, Rituximab‐treated group.

## 4 DISCUSSION

In this large, international cohort of patients with hematological malignancies receiving anti‐CD20 therapy, we identified a distinct pattern of increased COVID‐19 severity in those treated with obinutuzumab compared to those treated with rituximab. Despite being younger, having a lower comorbidity burden, and less aggressive HM, patients receiving obinutuzumab were at an increased risk of severe and critical COVID‐19 complications, including a higher hospitalization rate, ICU admission, and need for invasive mechanical ventilation.

Previous research has suggested that anti‐CD20 therapy might lead to a more severe course of COVID‐19 due to impaired humoral immunity and decreased antibody response to the virus or the vaccine.^9^, ^21^, ^22^ Our findings align with this hypothesis, adding to the body of knowledge by showing a differential impact between various anti‐CD20 therapies. While both obinutuzumab and rituximab lead to B‐cell depletion, obinutuzumab induces a more prolonged and profound depletion, which might have contributed to our cohort's increased severity of COVID‐19.

A recently published multicenter study on HM with FL performed in the pre‐COVID‐19 era (2016–2019) found no excess risk of infections among obinutuzumab patients in comparison to rituximab patients.^23^ The study demonstrated statistically similar viral infection rates among 21.7% of patients (rituximab group with 16/60 [26.7%] compared to 7/46 [15.2%]) in the obinutuzumab group (*p* = 1.0). Contrary to those results, in our cohort, among the subgroup of indolent lymphomas (*n* = 469), the incidence of COVID‐19 was 27.1%, with significantly higher rates of infection and increased severity in those treated with obinutuzumab. Whether the differences we observed reflect the unique pattern of SARS‐CoV‐2 immunomodulation or the higher infection rate during the pandemic is not apparent.

Current practice favors obinutuzumab treatment for FL despite no significant impact on overall survival rates. Although the GALLIUM study demonstrated an improved progression‐free survival (PFS) with obinutuzumab, adverse events, including infections and neutropenia, were more common. The rate of adverse events did not significantly differ between the induction and maintenance phases.^15^ The PRIMA study found a benefit in PFS without impacting overall survival when maintenance treatment with rituximab was compared to a placebo.^24^ We demonstrated an increased risk for severe outcomes in COVID‐19 illness in patients treated with obinutuzumab. Thus, we raise the question of whether clinicians should reconsider treatment regimens containing obinutuzumab in the presence of a respiratory viral pandemic.

Despite recent evidence showing a modest increase in humoral response to a third vaccine dose,^25^ we did not find that SARS‐CoV‐2 vaccination status or number of vaccine doses to be related to clinical outcomes. Furthermore, in line with previous studies,^10^ we found that T‐C monoclonal antibody prophylaxis was associated with a lower risk of severe COVID‐19, suggesting an essential role for this intervention in protecting patients receiving anti‐CD20 therapy. However, this option is currently unavailable, as these monoclonal antibodies have lost their neutralization ability against current variants.^26^, ^27^

Our study has some limitations, including its retrospective nature with the possibility of unmeasured confounding factors. Additionally, treatment with obinutuzumab is mainly used for HM with indolent lymphomas and CLL, creating an inherent difference between the two groups; thus, it was not included in our multivariate analysis. To address this limitation, we performed a subgroup analysis of the patients with indolent lymphomas or CLL, which shows a similar trend in ICU admissions and mechanical ventilation as the larger cohort (Table S2a,b). Another limitation when assessing outcomes as admission rates (hospitalization or ICU admission) in a multicenter study is the different site‐to‐site criteria for these events. To address this variance, we defined the primary outcome as the disease severity, which relies on objective parament of oxygen dependence, and mechanical ventilation as an additional secondary outcome. Moreover, HM status as remission or relapsed/refractory disease depends on the availability of PET–CT scans. During the pandemic, access to nonemergency tests was limited and prone to extreme variation between the different sites. Therefore, we elected not to rely on this variable and focused on HM treatment status as a more reliable marker for disease status. Finally, most patients (87.6% of the total cohort and 76.5% of COVID‐19‐positive patients) were from a single country, which can influence the ability to implement the results in other populations. Despite these limitations, our study represents, to the best of our knowledge, the largest cohort of patients with HM treated with anti‐CD20 infected with Omicron‐variant SARS‐CoV‐2.

In conclusion, our study suggests that patients with hematological malignancies receiving obinutuzumab are at a higher risk of severe and critical COVID‐19 than those treated with rituximab. While further studies are needed to understand the mechanistic differences between these therapies better, our findings highlight an aspect of the infectious complications potentially arising from a more severe immune suppression by a new, more potent treatment. In this regard, COVID‐19 may provide a signal that could apply to other respiratory viral infections.

## AUTHOR CONTRIBUTIONS

**Tali Shafat:** Conceptualization (lead); formal analysis (lead); methodology (lead); writing – original draft (lead); writing – review and editing (equal). **Daniel Grupel:** Conceptualization (equal); methodology (lead); project administration (equal); writing – original draft (lead); writing – review and editing (equal). **Tzvika Porges:** Conceptualization (equal); data curation (equal); methodology (equal); validation (equal); writing – review and editing (equal). **Ran Abuhasira:** Formal analysis (equal); writing – review and editing (equal). **Ana Belkin:** Data curation (equal); writing – review and editing (equal). **Ofir Deri:** Formal analysis (equal); writing – review and editing (equal). **Yonatan Oster:** Data curation (equal); writing – review and editing (equal). **Shadi Zahran:** Data curation (equal); writing – review and editing (equal). **Ehud Horwitz:** Data curation (equal); writing – review and editing (equal). **Netanel Horowitz:** Data curation (equal); writing – review and editing (equal). **Hazim Khatib:** Data curation (equal); writing – review and editing (equal). **Marjorie Vieira Batista:** Data curation (equal); writing – review and editing (equal). **Anita Cassoli Cortez:** Data curation (equal); writing – review and editing (equal). **Tal Brosh‐Nissimov:** Data curation (equal); writing – review and editing (equal). **Yafit Segman:** Data curation (equal); writing – review and editing (equal). **Linor Ishay:** Data curation (equal); writing – review and editing (equal). **Regev Cohen:** Data curation (equal); writing – review and editing (equal). **Alaa Atamna:** Data curation (equal); writing – review and editing (equal). **Amy Spallone:** Data curation (equal); writing – review and editing (equal). **Roy F. Chemaly:** Conceptualization (equal); supervision (equal); writing – review and editing (equal). **Juan Carlos Ramos‐Ramos:** Data curation (equal); writing – review and editing (equal). **Michal Chowers:** Data curation (equal); writing – review and editing (equal). **Evgeny Rogozin:** Data curation (equal); writing – review and editing (equal). **Noga Carmi Oren:** Data curation (equal); writing – review and editing (equal). **Şiran Keske:** Data curation (equal); writing – review and editing (equal). **Orit Wolfovitz Barchad:** Data curation (equal); writing – review and editing (equal). **Lior Nesher:** Conceptualization (lead); methodology (lead); supervision (lead); writing – review and editing (lead).

## CONFLICT OF INTEREST STATEMENT

The authors declare that they have no conflicts of interest with respect to this study. This study did not receive funding support.

## Supporting information


Data S1.

## Data Availability

Data are available upon reasonable request from the corresponding author.
