# Supplementary material for: An international multicenter study comparing COVID‐19 omicron outcomes in patients with hematological malignancies treated with obinutuzumab versus rituximab
Source: Cancer Med. 2024 Feb 24;13(3):e6997. doi: 10.1002/cam4.6997 (PMC10891459; doi:10.1002/cam4.6997)
Supplement: Supplementary file 1 — Data S1. [file CAM4-13-e6997-s001.docx]

Supplementary materials

Table S1-a: Distribution of study population according to country of origin:

|  | R-G  (n=762) | O-G  (n=286) | Total  (n=1048) |
| --- | --- | --- | --- |
| Israel, n (%) | 661 (86.7) | 257 (89.9) | 918 (87.6) |
| Brazil, n (%) | 71 (9.3) | 20 (7.0) | 91 (8.7) |
| Spain, n (%) | 18 (2.4) | 0 (0) | 18 (1.7) |
| United state, n (%) | 8 (1.0) | 8 (2.8) | 16 (1.5) |
| Turkey, n (%) | 4 (0.5) | 1 (0.3) | 5 (0.5) |

Abbreviations: R-G – Rituximab treated group, O-G – Obinutuzumab treated group, COVID-19=SARS-CoV-2 disease 2019

Table S1-b: Distribution of **COVID-19 positive** study population according to country of origin:

|  | R-G  (n=191) | O-G  (n=103) | Total  (n=294) |
| --- | --- | --- | --- |
| Israel, n (%) | 137 (71.7) | 88 (85.4) | 225 (76.5) |
| Brazil, n (%) | 24 (12.6) | 6 (5.8) | 30 (10.2) |
| Spain, n (%) | 18 (9.4) | 0 (0) | 18 (6.1) |
| United state, n (%) | 8 (4.2) | 8 (7.8) | 16 (5.4) |
| Turkey, n (%) | 4 (2.1) | 1 (1.0) | 5 (1.7) |

Abbreviations: R-G – Rituximab treated group, O-G – Obinutuzumab treated group, COVID-19=SARS-CoV-2 disease 2019

|  | **Severe-critical COVID-19** | **Hospitalization** | **ICU admission** | **Mechanical ventilation** |
| --- | --- | --- | --- | --- |
| Obinutuzumab therapy (with rituximab as reference) | 2.06 (1.10-3.89) | 2.26 (1.29-3.96) | 2.89 (1.08-7.78) | 5.78 (1.53-21.77) |
| Charlson comorbidity index (for each additional point) | 1.23 (1.09-1.38) | 1.13 (1.01-1.25) | 1.16 (0.97-1.39) | 1.20 (0.96-1.51) |
| Sex, female | 0.95 (0.52-1.74) | 1.15 (0.67-1.94) | 1.14 (0.43-3.00) | 0.67 (0.20-2.30) |
| Tixagevimab-cilgavimab Prophylaxis | 0.32 (0.10-1.03) | 0.50 (0.21-1.18) | 0.31 (0.04-2.46) | 0.43 (0.05-3.76) |
| SARS-CoV-2 vaccination status, 3-4 doses (with 0-2 doses as reference) | 1.44 (0.75-2.74) | 0.92 (0.53-1.60) | 1.27 (0.45-3.55) | 1.00 (0.28-3.59) |

Table S2: Multivariable analysis demonstrating the association between anti-CD20 treatment, patient characteristics, and COVID-19 outcomes.

Abbreviations: COVID-19=SARS-CoV-2 disease 2019, ICU=intensive care unit

Table S3: Association between anti-CD20 treatment and patients' characteristics and ICU admission in patients with indolent lymphoma / chronic lymphocytes leukemia.

| Variable | 0R (95% CI) |
| --- | --- |
| Obinutuzumab therapy (with rituximab as reference) | 4.62 (1.14-18.67) |
| Charlson comorbidity index (for each additional point) | 1.21 (0.97-1.52) |
| Induction therapy stage (with maintenance as reference) | 2.59 (0.86-7.75) |

Abbreviations: COVID-19=SARS-CoV-2 disease 2019, ICU=intensive care unit

Table S4-a: Outcomes of COVID-19 for patients infected during the induction treatment phase.

| Variable | R-G  (n=154) | O-G  (n=37) | P value |
| --- | --- | --- | --- |
| Severe-critical COVID-19, n (%) | 34 (22.1) | 11 (29.7) | 0.325 |
| Hospitalizations, n (%) | 52 (33.8) | 16 (43.2) | 0.280 |
| ICU admissions, n (%) | 10 (6.5) | 7 (18.9) | 0.017 |
| No respiratory support, n (%) | 118 (76.6) | 22 (59.5) | 0.034 |
| Mechanical ventilation, n (%) | 4 (2.6) | 6 (16.2) | 0.001 |
| COVID-19 related mortality, n (%) | 10 (6.5) | 4 (10.8) | 0.372 |
| All-cause mortality, n (%) | 16 (10.4) | 4 (10.8) | 0.940 |

Abbreviations: R-G – Rituximab treated group, O-G – Obinutuzumab treated group, COVID-19=SARS-CoV-2 disease 2019, ICU=intensive care unit,

Table S4-b: Outcomes of COVID-19 for patients infected during the maintenance treatment phase.

| Variable | R-G  (n=31) | O-G  (n=63) | P value |
| --- | --- | --- | --- |
| Severe-critical COVID-19, n (%) | 8 (25.8) | 21 (33.3) | 0.458 |
| Hospitalizations, n (%) | 13 (41.9) | 37 (58.7) | 0.125 |
| ICU admissions, n (%) | 1 (3.3) | 6 (9.5) | 0.422 |
| No respiratory support, n (%) | 23 (74.2) | 42 (66.7) | 0.458 |
| Mechanical ventilation, n (%) | 0 (0.0) | 5 (7.9) | 0.167 |
| COVID-19 related mortality, n (%) | 1 (3.2) | 6 (9.5) | 0.419 |
| All-cause mortality, n (%) | 1 (3.2) | 6 (9.5) | 0.419 |

Abbreviations: R-G – Rituximab treated group, O-G – Obinutuzumab treated group, COVID-19=SARS-CoV-2 disease 2019, ICU=intensive care unit,
